# Time trends in the use of curative treatment in men 70 years and older with nonmetastatic prostate cancer

**DOI:** 10.2340/1651-226X.2024.26189

**Published:** 2024-03-20

**Authors:** Frida E Lundberg, David Robinson, Ola Bratt, Giuseppe Fallara, Mats Lambe, Anna L. V. Johansson

**Affiliations:** a Department of Medical Epidemiology and Biostatistics, Karolinska Institutet, Stockholm, Sweden; b Department of Oncology-Pathology, Karolinska Institutet, Stockholm, Sweden; c Department of Urology, Ryhov Hospital, Jönköping, Sweden; d Department of Urology, Sahlgrenska University Hospital, Gothenburg, Sweden; e Department of Urology, IRCCS IEO European Institute of Urology, Milan, Italy; f Regional Cancer Center Central Sweden, Uppsala, Sweden; g Cancer Registry of Norway, Oslo, Norway

**Keywords:** Prostate cancer, management, age, mortality, Sweden

## Abstract

**Background:**

Undertreatment of otherwise healthy men in their seventies with prostate cancer has been reported previously.

**Material and methods:**

Using information in a Swedish prostate cancer research database, patterns of management and cancer-specific mortality were compared across age groups in over 70,000 men diagnosed with intermediate- or high-risk nonmetastatic prostate cancer between 2008 and 2020. Crude probabilities of death were estimated non-parametrically. Staging procedures, primary treatment, and cancer death were compared using regression models, adjusting for patient and tumor characteristics.

**Results:**

During the study period, the proportion of men treated with curative intent increased in ages 70–74 (intermediate-risk from 45% to 72% and high-risk from 49% to 84%), 75–79 (intermediate-risk from 11% to 52% and high-risk from 12% to 70%), and 80–84 years (intermediate-risk from < 1% to 14% and high-risk from < 1% to 30%). Older age was associated with lower likelihoods of staging investigations and curative treatment, also after adjustment for tumor characteristics and comorbidity. Men treated with curative intent and those initially managed conservatively had lower crude risks of prostate cancer death than men receiving androgen deprivation treatment (ADT). In adjusted analyses, ADT was associated with higher prostate cancer mortality than curative treatment across ages and risk groups. Among men managed conservatively, prostate cancer mortality was higher in ages 70 and above.

**Interpretation:**

Use of curative treatment increased substantially in older men with prostate cancer between 2008 and 2020. Our findings suggest reduced age-bias and under-treatment, likely reflecting improved individualized decision-making and adherence to guidelines recommending more active management of older men.

## Introduction

Studies conducted in different settings have found evidence of medically unjustified differences in patterns of care between younger and older cancer patients [1–5]. Although a lower staging and treatment activity may be justified by a high comorbidity burden, frailty, and a short life expectancy, management decisions are also driven by chronological age [5–7]. There may also be differences in the uptake of new diagnostic and therapeutic methods across age groups. In addition, defining the optimal management of cancer in older age groups is hampered by underrepresentation of older patients in clinical studies.

Several studies have found evidence of undertreatment of otherwise healthy men in their seventies with intermediate or high-risk nonmetastatic prostate cancer [1, 2, 8, 9]. In an earlier Swedish study, it was also noted that treatment with curative intent had increased in all age groups between 2001 and 2012 [1]. Findings from another recent Swedish study, investigating the association between age at diagnosis and prostate cancer prognosis, found indirect evidence of insufficient staging and curative treatment in older men [10].

The number of healthy older individuals without a history of serious illness or risk factors such as smoking has increased and is expected to continue to increase [6, 11]. In 2021, the mean remaining life expectancy in Sweden was more than 10 years for a 77-year-old man and exceeded 5 years for a man aged 87 [12]. Similar to recommendations by the American Urological Association [13] and the European Association of Urology [14], Swedish prostate cancer guidelines emphasize the importance of incorporating health status and life expectancy in clinical decision-making [15]. However, converging evidence indicates that adherence to guidelines often is suboptimal, with life expectancy and comorbidity burden being poorly incorporated in decision-making [1, 10, 16].

By use of nationwide registers, including detailed clinical information, we aimed to investigate whether patterns of staging and treatment differed in older and younger men with intermediate or high-risk localized prostate cancer and to assess changes over time. We chose to focus on men in ages 70–79 years, who have an estimated life expectancy of approximately 10–15 years. We also examined whether there were differences in 10-year cancer mortality by treatment modality across age groups.

## Material and methods

### Data and study population

In Sweden, a total of 130,874 men/cases were diagnosed with prostate cancer between 2008 and 2020 and included in the Swedish National Prostate Cancer Register (NPCR), of which 26.0% were low-risk, 33.1% intermediate risk, 20.6% high-risk nonmetastatic, 5.5% regional metastatic, 11.8% distant metastatic, and 3.0% of unknown risk group [17].

The NPCR includes detailed information on tumor characteristics, diagnostic and staging procedures, and planned primary treatment of 98% of all men diagnosed with prostate cancer in Sweden since 1998. By use of the personal identification number assigned to all Swedish residents, NPCR data have been cross-linked with other national registers to create the Prostate Cancer Data Base Sweden (PCBaSe 5.0) [18]. These registers include the Swedish Cancer Register, the National Patient Register, the Prescribed Drug Register, the Population Register, the Cause of Death Register, and the Longitudinal Integration Database for Insurance and Labour Market Studies (LISA).

This cohort study included all men registered in PCBaSe 5.0, who were diagnosed with intermediate- or high-risk nonmetastatic prostate cancer between 2008 and 2020 (*N* = 70,074).

PCBaSe 5.0 also includes a comparison population of five men free of prostate cancer per cancer case, randomly selected from the Population Register after matching on birth-year and county of residence at the time of the corresponding case’s diagnosis. We used information from PCBaSe 5.0 on patient characteristics, including comorbidity assessed by the Charlson Comorbidity Index (CCI) [19, 20] and a Drug Comorbidity Index (DCI) [21], marital status, educational level, region of birth, tumor characteristics, diagnostic and staging procedures, treatment, and follow-up, including date and cause of death and emigration. We also obtained information on comorbidity among men in the comparison population free of prostate cancer.

### Cancer characteristics

Data on cancer characteristics included local tumor stage (T), clinical lymph node involvement (N), distant metastasis (M), prostate-specific antigen (PSA) value, and Gleason score at diagnosis. Based on this information, only patients with intermediate-risk (T1–2, Gleason score 7 or PSA 10–19 ng/mL, Nx/N0, Mx/M0) and high-risk nonmetastatic disease (T3 or Gleason score 8–10 or PSA 20–49 ng/mL, Nx/N0, Mx/M0) were included.

### Staging procedures

We analyzed the proportion of men with high-risk prostate cancer who had undergone abdominal imaging (magnetic resonance imaging [MRI], computerized tomography [CT], or positron emission tomography-computed tomography [PET-CT]) and bone imaging (MRI, CT, PET-CT, X-ray, or bone scintigraphy). The Swedish guidelines recommended bone imaging before curative treatment of high-risk disease throughout the study period; abdominal imaging was not recommended until 2017.

### Treatment

Planned primary treatment was categorized into curative (radical prostatectomy or radiotherapy), conservative (active surveillance or watchful waiting), or androgen deprivation therapy (ADT: gonadotropin releasing hormones, antiandrogens, or orchiectomy).

### Comorbidity

For each man, the comorbidity burden was estimated by use of CCI and DCI. The CCI was calculated based on diagnoses recorded in the National Inpatient Register up to 15 years before prostate cancer diagnosis. The DCI was based on drug dispensations recorded in the Prescribed Drug Register up to 1 year before diagnosis as described by Gedeborg et al. [21].

### Statistical methods

Six age groups were analyzed (< 65, 65–69, 70–74, 75–79, 80–84, and ≥ 85 years at diagnosis), with men aged 65–69 assigned as the reference group in all analyses. Odds ratios (ORs) with 95% confidence intervals (CIs) for associations between age group and use of staging procedures were estimated using logistic regression. Relative risk ratios (RRRs) with 95% CIs for receiving curative treatment versus conservative management or ADT were estimated using multinomial regression. Regression models were stepwise adjusted for patient factors, tumor characteristics, and finally comorbidity (CCI and DCI). Associations between age and primary treatment planned were also estimated separately for both men with CCI 0 and those with CCI 0 and DCI 1st quartile. Within each age group, the distributions of CCI and DCI were compared with those in the control population using Chi-square tests. To investigate changes over time, associations between age and primary treatment planned were estimated separately for two calendar periods (2008–2016 and 2017–2020). From 2015, Swedish guidelines have recommended increased use of curative treatment in older men with high-risk prostate cancer.

Survival time was defined from the date of diagnosis until prostate cancer death, death from other cause, emigration, or up to 10 years of follow-up, whichever came first. Crude probabilities of prostate cancer death and other cause death were estimated using non-parametric competing risks methods (Stata command stcompet). Associations between age, primary treatment, and cancer-specific mortality were assessed by mortality rate ratios (MRRs) with 95% CIs estimated by Cox proportional hazards models. The models included the interaction between age group and treatment type (curative, conservative, or ADT) with stepwise adjustment for patient and tumor characteristics and comorbidity (CCI and DCI). Interactions were tested using the likelihood ratio test, and the proportional hazards assumption was assessed using tests based on Schoenfeld residuals. Based on these tests, we concluded that the proportional hazards assumption was fulfilled. Analyses were performed using Stata MP/16.1 (Stata Statistical Software: Release 16; Stata Corp LLC, College Station, Texas, 2019).

## Results

Between 2008 and 2020, a total of 43,229 men were diagnosed with intermediate-risk prostate cancer and 26,845 with high-risk prostate cancer (Supplementary Table 1 and Supplementary Table 2). Within both risk groups, older men had higher stage PSA values and Gleason scores at diagnosis. In men with intermediate-risk prostate cancer, 36% were diagnosed in age group 70–79 years and 7.2% in men 80 years or older. The corresponding proportions in men with high-risk disease were 41% (70–79) and 24% (≥ 80).

The comorbidity burden increased with age in both risk groups, as estimated by both CCI and DCI (Supplementary Figure 1 and Supplementary Figure 2). There were only small absolute differences in CCI between risk groups within 5-year age groups. In all age groups, men with prostate cancer had higher drug comorbidity, i.e. they were less likely to have a DCI in the first (lowest) quartile compared to control men.

### Staging procedures

**Figure 1:**
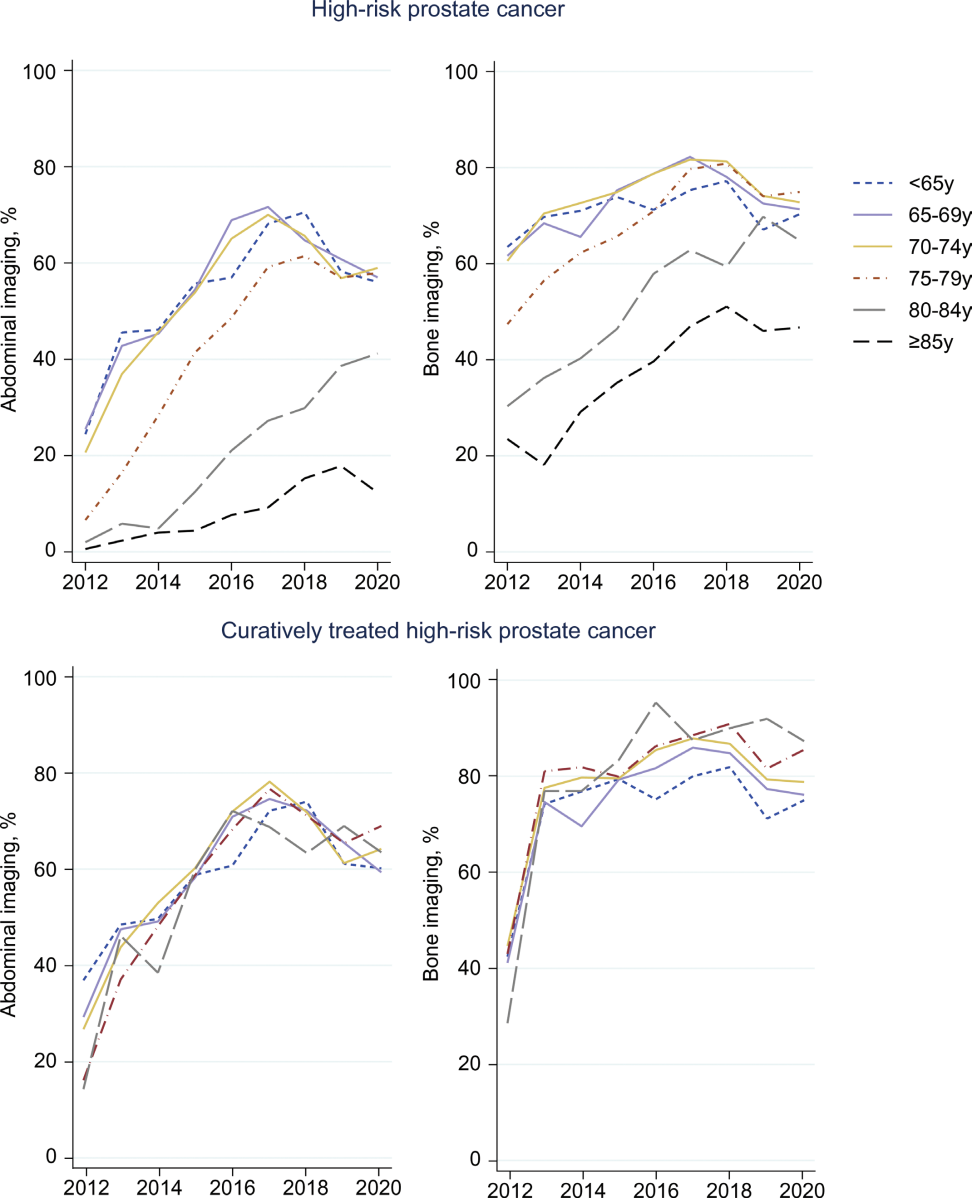
Proportion of patients with high-risk prostate cancer undergoing abdominal and bone imaging over time.

In men with high-risk disease, the likelihood of undergoing abdominal and bone imaging was lower in men 75–79 years (44%) than in those aged 65–69 (54%) and was particularly low in men ≥ 80 years (80–84: 20% and ≥ 85: 8%) (Supplementary Table 3). The likelihood of undergoing bone imaging was lower in men aged 80 and above (80–84: 52% and ≥ 85: 38%), and around 70% in all other age groups. Between 2012 and 2020, the proportion of patients undergoing abdominal imaging increased in all age groups, but with a later uptake in the older age groups (Figure 1, first row). Bone imaging also became more common over time, especially among men aged 75 and above. However, of note was evidence of slight a decline from around 2017 in the use of both abdominal and bone imaging in men below age 80 years. When restricting to men who received treatment with curative intent, the proportions of men undergoing diagnostic abdominal and bone imaging were similar between older and younger age groups (Figure 1, second row). Among the 17 men aged ≥ 85 who received curative treatment for high-risk disease between 2012 and 2020, 53% had abdominal imaging and 76% had bone imaging.

### Treatment

**Figure 2:**
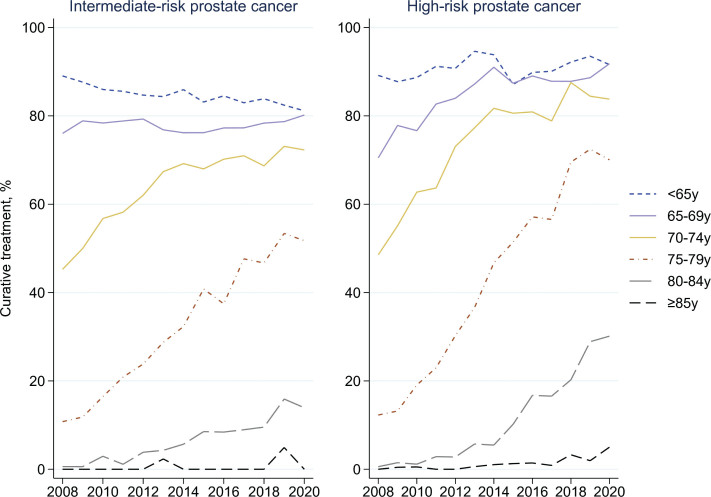
Proportion of patients with intermediate- and high-risk prostate cancer treated with curative intent over time.

In both risk groups and across age groups, the proportion of men over 65 years treated with curative intent increased between 2008 and 2020 (Figure 2). The most marked increase was observed in men ages 75–79 years: from 11% to 52% in men with intermediate-risk and from 12% to 70% in men with high-risk cancer. The proportion of men aged 70–74 years treated with curative intent also increased substantially (intermediate-risk 45%–72% and high-risk 49%–84%), almost reaching that in younger age groups in 2020. Also in men aged 80–84 years, there was a substantial increase in curative treatments, from nearly none (< 1%) in 2008 to 14% (intermediate-risk) and 30% (high-risk) in 2020.

During the entire period under study, the proportion of men receiving curative treatment decreased with age, while conservative management and ADT treatment became increasingly common, both in men with intermediate- and high-risk prostate cancer (Table 1). In multinomial regression models comparing the likelihood of treatment with curative intent versus conservative management or ADT, the likelihood of curative treatment decreased with increasing age. In men with intermediate-risk prostate cancer, the estimated RRRs ranged from 0.58 (0.54–0.61) in age group 70–74 years to 0.03 (0.03–0.04) in men 80–84 years, compared with the reference group (men ages 65–69, Model 1). Stepwise adjustments for tumor characteristics (Model 2) and comorbidity (Model 3) did not substantially change these estimates. The adjusted RRRs of curative treatment vs ADT also decreased with age.

**Table 1:** Primary treatment planned for intermediate- and high-risk prostate cancer patients.

			RRR comparing curative to conservative and ADT		
Intermediate-risk prostate cancer			Model 1	Model 2	Model 3
**Curative vs conservative**	**Curative**	**Cons.**	**RRR (95% CI)**	**RRR (95% CI)**	**RRR (95% CI)**
< 65 y	11,099 (85%)	1,946 (15%)	1.53 (1.43–1.64)	1.58 (1.46–1.69)	1.49 (1.39–1.61)
65–69 y	8,545 (78%)	2,258 (21%)	1.00 (reference)	1.00 (reference)	1.00 (reference)
70–74 y	6,274 (65%)	2,817 (29%)	0.58 (0.54–0.61)	0.53 (0.49–0.56)	0.55 (0.51–0.59)
75–79 y	2,088 (36%)	2,649 (46%)	0.20 (0.19–0.22)	0.16 (0.15–0.17)	0.17 (0.16–0.18)
80–84 y	161 (7%)	1,340 (58%)	0.03 (0.03–0.04)	0.02 (0.02–0.03)	0.02 (0.02–0.03)
≥ 85 y	4 (1%)	398 (56%)	0.00 (0.00–0.00)	0.00 (0.00–0.00)	0.00 (0.00–0.00)
**Curative vs ADT**	**Curative**	**ADT**			
< 65 y	11,099 (85%)	67 (1%)	3.52 (2.65–4.68)	3.44 (2.58–4.59)	3.06 (2.29–4.09)
65–69 y	8,545 (78%)	175 (2%)	1.00 (reference)	1.00 (reference)	1.00 (reference)
70–74 y	6,274 (65%)	500 (5%)	0.24 (0.20–0.29)	0.25 (0.21–0.30)	0.27 (0.22–0.32)
75–79 y	2,088 (36%)	1,083 (19%)	0.03 (0.03–0.04)	0.04 (0.03–0.04)	0.04 (0.03–0.05)
80–84 y	161 (7%)	805 (35%)	0.00 (0.00–0.00)	0.00 (0.00–0.01)	0.01 (0.00–0.01)
≥ 85 y	4 (1%)	312 (44%)	0.00 (0.00–0.00)	0.00 (0.00–0.00)	0.00 (0.00–0.00)
**High-risk prostate cancer**			**Model 1**	**Model 2**	**Model 3**
**Curative vs conservative**	**Curative**	**Cons.**	**RRR (95% CI)**	**RRR (95% CI)**	**RRR (95% CI)**
< 65 y	3,837 (91%)	235 (6%)	1.39 (1.16–1.66)	1.68 (1.37–2.05)	1.57 (1.29–1.93)
65–69 y	4,060 (85%)	323 (7%)	1.00 (reference)	1.00 (reference)	1.00 (reference)
70–74 y	4,139 (74%)	508 (9%)	0.58 (0.50–0.67)	0.50 (0.42–0.59)	0.52 (0.44–0.62)
75–79 y	2,301 (43%)	775 (15%)	0.19 (0.16–0.22)	0.13 (0.11–0.16)	0.15 (0.12–0.17)
80–84 y	412 (10%)	713 (18%)	0.03 (0.03–0.04)	0.02 (0.02–0.03)	0.02 (0.02–0.03)
≥ 85 y	28 (1%)	497 (20%)	0.00 (0.00–0.00)	0.00 (0.00–0.00)	0.00 (0.00–0.00)
**Curative vs ADT**	**Curative**	**ADT**			
< 65 y	3,837 (91%)	160 (4%)	2.63 (2.17–3.19)	2.65 (2.18–3.23)	2.46 (2.02–3.01)
65–69 y	4,060 (85%)	413 (9%)	1.00 (reference)	1.00 (reference)	1.00 (reference)
70–74 y	4,139 (74%)	920 (17%)	0.40 (0.35–0.45)	0.40 (0.35–0.46)	0.42 (0.37–0.49)
75–79 y	2,301 (43%)	2,230 (42%)	0.08 (0.07–0.09)	0.08 (0.07–0.09)	0.09 (0.08–0.10)
80–84 y	412 (10%)	2,816 (71%)	0.01 (0.01–0.01)	0.01 (0.01–0.01)	0.01 (0.01–0.01)
≥ 85 y	28 (1%)	1,906 (78%)	0.00 (0.00–0.00)	0.00 (0.00–0.00)	0.00 (0.00–0.00)

Model 1: Adjusted for marital status, education, country of birth, and year of diagnosis.

Model 2: Also adjusted for tumor size, Gleason sum, and PSA-level.

Model 3: Also adjusted for Charlson Comorbidity Index and Drug Comorbidity Index.

ADT: androgen deprivation therapy; CI: confidence interval; Cons: conservative; PSA: prostate-specific antigen; RRR: relative risk ratio.

Similar associations were observed in men with high-risk prostate cancer. Compared with men aged 65–69 years, the likelihood of receiving curative treatment versus conservative management was markedly lower in men aged 70–74 years (RRR = 0.58; 0.50–0.67), aged 75–79 years (RRR = 0.19; 0.16–0.22), and in men aged 80–84 years (RRR = 0.03; 0.03–0.04) (Model 1), associations which remained following adjustments for tumor characteristics and comorbidity (Models 2 and 3). Men over 80 years were also much more likely to receive ADT than curative treatment. Further adjustments had very minor influence on these associations.

In separate analyses restricted to men with the lowest comorbidity burden (either CCI 0 or both CCI 0 and DCI 1st quartile), the associations between age and the likelihood to receive curative intent treatment were similar to the overall estimates in Table 1, both in men with intermediate- and high-risk prostate cancer (Supplementary Table 4 and Supplementary Table 5).

In analyses stratified by calendar period, the age-differences in the likelihood of receiving curative treatment were more pronounced in the earlier period (2008–2016) but remained significant also for men diagnosed between 2017 and 2020 (Supplementary Table 6). The exception was men with high-risk prostate cancer aged 70–74 compared to 65–69 (adjusted RRR 0.79, 95% CI 0.56–1.10).

### Prostate cancer-specific mortality

**Figure 3:**
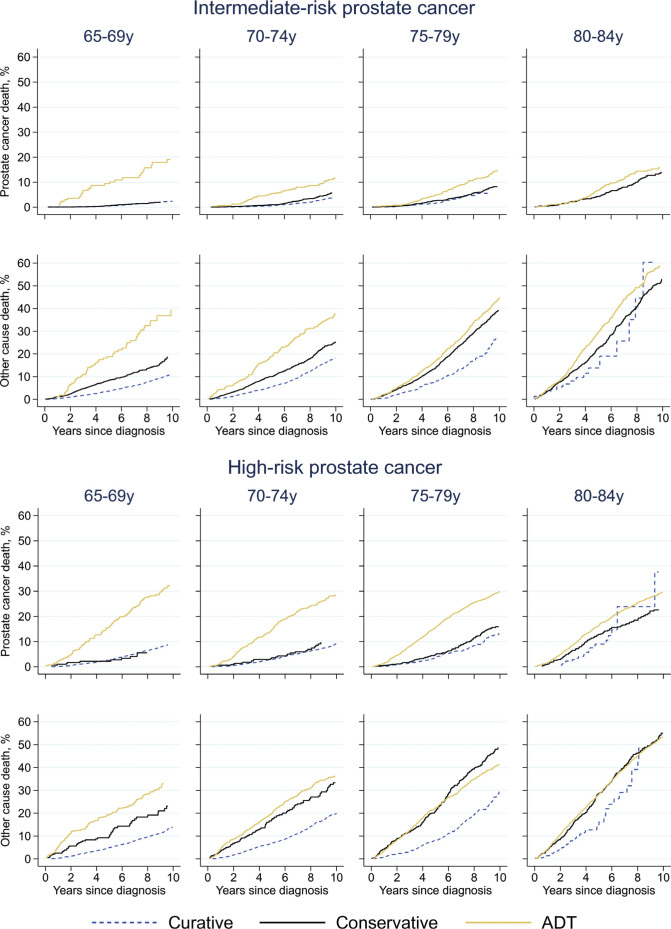
Crude probability of death due to prostate cancer and other causes. ADT = androgen deprivation therapy. Curatively treated intermediate-risk prostate cancer aged 80-84 years: prostate cancer death not shown in graph due to only 2 events.

In men with intermediate-risk cancer, the crude probability of prostate cancer death was relatively low: < 10% at 5 years (Figure 3). However, in men aged 65–69 years, the crude probability was substantially higher in those planned for primary ADT than in the men with planned curative treatment or conservative management, particularly in men with high-risk disease. Other-cause mortality was also generally higher in men receiving ADT in men below 75 years. In men with high-risk disease, ADT was associated with higher crude probabilities of prostate cancer death in men below 80 years, while the probability was similar in men planned for curative or conservative management. Other-cause mortality was lower in men treated with curative intent than in men initially receiving conservative treatment or ADT.

In adjusted analyses, prostate cancer-specific mortality increased with age within all treatment groups, compared with men aged 65–69 years planned for curative treatment (Table 2). Men with intermediate-risk cancer who were initially managed conservatively had similar cancer-specific mortality as men planned for curative treatment in ages below 70, but higher mortality in age groups 70–74 (HR = 1.8, 95% CI 1.3–2.5) and 75–79 years (HR = 1.5, 95% CI 1.0–2.3). Men with intermediate-risk tumors planned for ADT had higher mortality than curatively treated men of the same age in all ages, although not statistically significant in age group 80–84. In men aged 70 and above with high-risk cancer, conservative management was associated with higher mortality than curative treatment. Furthermore, prostate cancer-specific mortality was significantly higher in men planned for ADT compared with those planned for curative treatment in all age groups.

**Table 2:** Cancer-specific mortality and intermediate- and high-risk prostate cancer patients up to 84 years.

Intermediate-risk prostate cancer	Cancer deaths			MRR (95% CI)		
**Between age groups**	**Cur.**	**Cons.**	**ADT**	**Curative**	**Cons.**	**ADT**
< 65 y	75	10	9	0.6 (0.5–0.9)	0.7 (0.3–1.3)	8.5 (4.2–17.0)
65–69 y	93	21	25	1.0 (reference)	1.1 (0.7–1.8)	8.8 (5.6–14.1)
70–74 y	79	68	43	1.4 (1.1–1.9)	2.6 (1.9–3.5)	4.7 (3.2–7.0)
75–79 y	33	99	96	2.7 (1.8–4.0)	4.1 (3.0–5.4)	6.2 (4.6–8.4)
80–84 y	2	92	71	3.2 (0.8–13.2)	7.1 (5.2–9.7)	7.9 (5.6–11.1)
**Within each age group**	**Cur.**	**Cons.**	**ADT**	**Curative**	**Cons.**	**ADT**
< 65 y	75	10	9	1.0 (reference)	1.1 (0.5–2.0)	13.4 (6.7–27.1)
65–69 y	93	21	25	1.0 (reference)	1.1 (0.7–1.8)	8.8 (5.6–14.1)
70–74 y	79	68	43	1.0 (reference)	1.8 (1.3–2.5)	3.3 (2.2–4.9)
75–79 y	33	99	96	1.0 (reference)	1.5 (1.0–2.3)	2.3 (1.5–3.5)
80–84 y	2	92	71	1.0 (reference)	2.2 (0.5–9.0)	2.4 (0.6–10.0)
High-risk prostate cancer	Cancer deaths			MRR (95% CI)		
**Between age groups**	**Cur.**	**Cons.**	**ADT**	**Curative**	**Cons.**	**ADT**
< 65 y	186	6	64	1.0 (0.8–1.3)	1.3 (0.6–3.0)	6.1 (4.5–8.2)
65–69 y	186	11	110	1.0 (reference)	1.5 (0.8–2.8)	4.6 (3.6–5.8)
70–74 y	166	27	208	1.0 (0.8–1.3)	1.8 (1.2–2.9)	4.1 (3.3–5.0)
75–79 y	89	80	498	1.3 (1.0–1.7)	3.2 (2.4–4.2)	4.6 (3.9–5.5)
80–84 y	18	109	559	2.3 (1.4–3.8)	5.2 (4.0–6.8)	4.9 (4.1–5.8)
**Within each age group**	**Cur.**	**Cons.**	**ADT**	**Curative**	**Cons.**	**ADT**
< 65 y	186	6	64	1.0 (reference)	1.3 (0.6–2.9)	5.8 (4.4–7.9)
65–69 y	186	11	110	1.0 (reference)	1.5 (0.8–2.8)	4.6 (3.6–5.8)
70–74 y	166	27	208	1.0 (reference)	1.8 (1.1–2.8)	4.0 (3.2–4.9)
75–79 y	89	80	498	1.0 (reference)	2.4 (1.7–3.3)	3.5 (2.8–4.4)
80–84 y	18	109	559	1.0 (reference)	2.3 (1.3–3.8)	2.1 (1.3–3.4)

Adjusted for marital status, country of birth, education level, year of diagnosis, tumor size, Gleason sum, PSA-level, CCI, and DCI. ADT: androgen deprivation therapy, CCI: Charlson Comorbidity Index, CI: confidence interval, Cons.: Conservative, Cur.: Curative, PSA: prostate-specific antigen, DCI: Drug Comorbidity Index, MRR: mortality rate ratio. Ages ≥ 85 years were excluded from the analyses since there were no events among curatively treated.

## Discussion

This large population-based Swedish study found age-differences in the management and cancer-specific survival in men with intermediate- and high-risk prostate cancer during the first time-period under study. Use of curative treatment increased dramatically over time in men aged ≥ 70 years, albeit less so among the oldest (≥ 80 years). Our results, thus, indicate a reduction in undertreatment in older men during recent years with diminishing age-differences. During the entire study period, one-fifth of men aged 75–79 years with intermediate-risk prostate cancer received ADT instead of curative treatment compared to less than 5% in younger men. Between 2017 and 2020, the corresponding proportions were 10% and < 2%. The use of abdominal and bone imaging increased in all age-groups but remained markedly lower in men above 84 years. The proportion of older men with high-risk disease who were properly staged before treatment with curative intent was, however, similar to the proportions in younger men. This proportion declined in 2019 and 2020, which was probably related to the COVID-19 pandemic in 2020, while we have no explanation for the decline in 2019.

According to current Swedish guidelines [15], treatment with curative intent is not indicated in men with intermediate-risk cancer who have a life expectancy shorter than 10–15 years or in men with high-risk cancer who have a life expectancy shorter than 5–10 years. Based on current estimates of life expectancy [12], most Swedish men up to 75 years with intermediate-risk cancer and some men up to 85 years with high-risk cancer should be considered and evaluated as candidates for curative treatment. However, in analyses adjusted for comorbidity and other potentially modifying factors, we found that both men with intermediate- and men with high-risk prostate cancer aged 70–79 years (with an expected life expectancy of about 10–15 years) were less likely to receive curative treatment than younger men. We found only small differences in comorbidity burden in men with prostate cancer compared to controls, suggesting a similar life expectancy as in that the background population.

Our results from the start of the study period in 2008 until the mid-2010s corroborate earlier reports of undertreatment of older men. Several investigators have concluded that treatment decisions in older patients appear to often be made without proper consideration of general health status [22–26] and life expectancy [1, 8, 22, 24]. In a Dutch nationwide register-based study, older men with intermediate- or high-risk prostate cancer less often received treatment with curative intent also after consideration of tumor characteristics, PSA-levels, and comorbidity burden [2]. Similarly, in analyses adjusted for comorbidity, an Australian study including men 70 years and older with localized prostate cancer found that men in the oldest age group (80–89 years) were significantly less likely to receive curative treatment [9]. Also, Lunardi et al. found that in France, general health status was not considered in clinical decision making, resulting in undertreatment of older and overtreatment of younger men with low-risk prostate cancer [8]. Bratt et al. reported that otherwise healthy Swedish men with high-risk cancer in their 70s were less likely to get curative treatment than younger men with similar life expectancy [1].

Importantly, we observed increasing use of curative treatment over time in all age groups above 70 years, however, with a remaining lower use in the oldest men (≥ 80 years). This trend is likely to reflect recommendations of more active treatment across age groups in the Swedish national guidelines for the management of high-risk prostate cancer [15].

The much higher cancer-specific mortality within age groups after initial conservative management or ADT compared with curative treatment suggests that undertreatment was common. As use of curative treatment increased during the studied time period, undertreatment may have been an issue mainly or only in the early years. There was evidence of undertreatment during the first part of the study period also in men with a remaining life expectancy of more than 10 years, also following adjustments for tumor characteristics, educational level, and comorbidity burden. As expected, prostate cancer-specific mortality increased with age both in curatively and non-curatively treated men. In both risk groups and within each age group, the highest mortality was observed in men receiving ADT. This was particularly apparent in men aged 65–69 years who received ADT only. This mortality-gap may in part be explained by unmeasured confounding, e.g. performance status or general frailty in men receiving ADT.

In a Surveillance, Epidemiology, and End Results (SEER) study encompassing more than half a million men with a prostate cancer diagnosis between 2004 and 2016, older age was not associated with higher cancer-specific mortality in men with intermediate- or high-risk prostate cancer [27]. However, in that study, no information was available on patterns of treatment or comorbidity burden. A Dutch register-based study including almost 100,000 men with a prostate cancer diagnosis between 2005 and 2015 found a poorer survival in older compared to younger men [2]. These differences remained after adjustment for PSA levels, tumor characteristics, and comorbidities and were most pronounced in men with localized high-risk prostate cancer. However, survival of men treated with curative intent was similar across age groups. Based on analyses of more than 40,000 men with a diagnosis of localized prostate cancer 2004–2014 identified in the SEER database, Bandini et al. found a lower mortality in men ≥ 75 years who had undergone radical prostatectomy or received radiotherapy compared those receiving nonlocal treatment [28]. Similarly, in a Swedish study of 121,000 men aged 55–95 years diagnosed with prostate cancer between 1998 and 2012, older age was associated with higher prostate cancer mortality after adjustment for tumor characteristics, mode of detection, treatment, and comorbidity [10].

Strengths of our study included the nation-wide, population-based setting with detailed information available on clinical factors and aspects of management, tumor characteristics, and first line treatment. Including more than 70,000 men, this is one of the largest studies to date addressing the role of age in the management of men with intermediate- and high-risk prostate cancer. Individual level record linkages with data retrieved from several nationwide registers provided information on potential confounding factors and a virtually complete follow-up. Since our estimation of comorbidity by use of the CCI was based on records of in-hospital care, the group of men categorized as having no comorbidity (CCI 0) could include both healthy individuals and those with concomitant medical conditions cared for outside a hospital setting. However, we were also able to include DCI using data from the Prescribed Drug Register to capture conditions often managed in primary care settings, such as arrhythmias, hyperlipidemia, and diabetes.

Several limitations need mentioning. No information was available on performance status or general frailty, cognitive function, nutritional status, functional status, smoking, or dependence. Thus, the data at hand could not capture all factors needed for a thorough assessment of an individual’s life expectancy. In addition, the role of physician versus patient preferences on choice of treatment could not be assessed. Information on diagnostic imaging was unavailable before 2012, and records of prostate MRI were not available before 2015. Similar to other observational studies in this area, selection and confounding by indication is a concern, hampering the interpretation of results, particularly regarding patterns of cancer-specific mortality. Also, direct comparisons to results of other studies are potentially hampered by difficulties in defining undertreatment, particularly in older men where there is a challenge to determine the optimal choice of management in a risk-benefit perspective. Although our study is a nation-wide and population-based study, the findings may not be generalizable to other populations with different life-expectancy and comorbidity patterns. Finally, information on cause of death is not always reliable, especially in older patients where prostate cancer can incorrectly be assigned as the cause of death [29].

## Conclusions

The use of curative treatment increased substantially in men aged 70–84 years with intermediate- or high-risk prostate cancer. Our findings indicate that undertreatment of older men with prostate cancer was common in Sweden up until recent years. The increased use of curative treatment likely reflects adherence to national guidelines, including recommendations of more active treatment of older men and an improved awareness of the importance to avoid age-biased clinical decision-making. The individual decision whether to recommend curative treatment to older men and to men with comorbidities is, however, difficult. The ongoing Scandinavian randomized clinical trial GrandP/SPCG-19 (NCT05448547) investigates this question in men over 75 years with high-risk nonmetastatic prostate cancer.

## Supplementary Material

Time trends in the use of curative treatment in men 70 years and older with nonmetastatic prostate cancer

Time trends in the use of curative treatment in men 70 years and older with nonmetastatic prostate cancer
