# Supplementary material for: Time trends in the use of curative treatment in men 70 years and older with nonmetastatic prostate cancer
Source: Acta Oncol. 2024 Mar 20;63:26189. doi: 10.2340/1651-226X.2024.26189 (PMC11332516; doi:10.2340/1651-226X.2024.26189)
Supplement: Time trends in the use of curative treatment in men 70 years and older with nonmetastatic prostate cancer [file AO-63-26189-s1.pdf]

Supplementary material has been published as submitted. It has not been copyedited or typeset by Acta Oncologica.

Supplementary Figure 1. Charlson Comorbidity Index in intermediate- and high-risk prostate cancer cases compared to controls

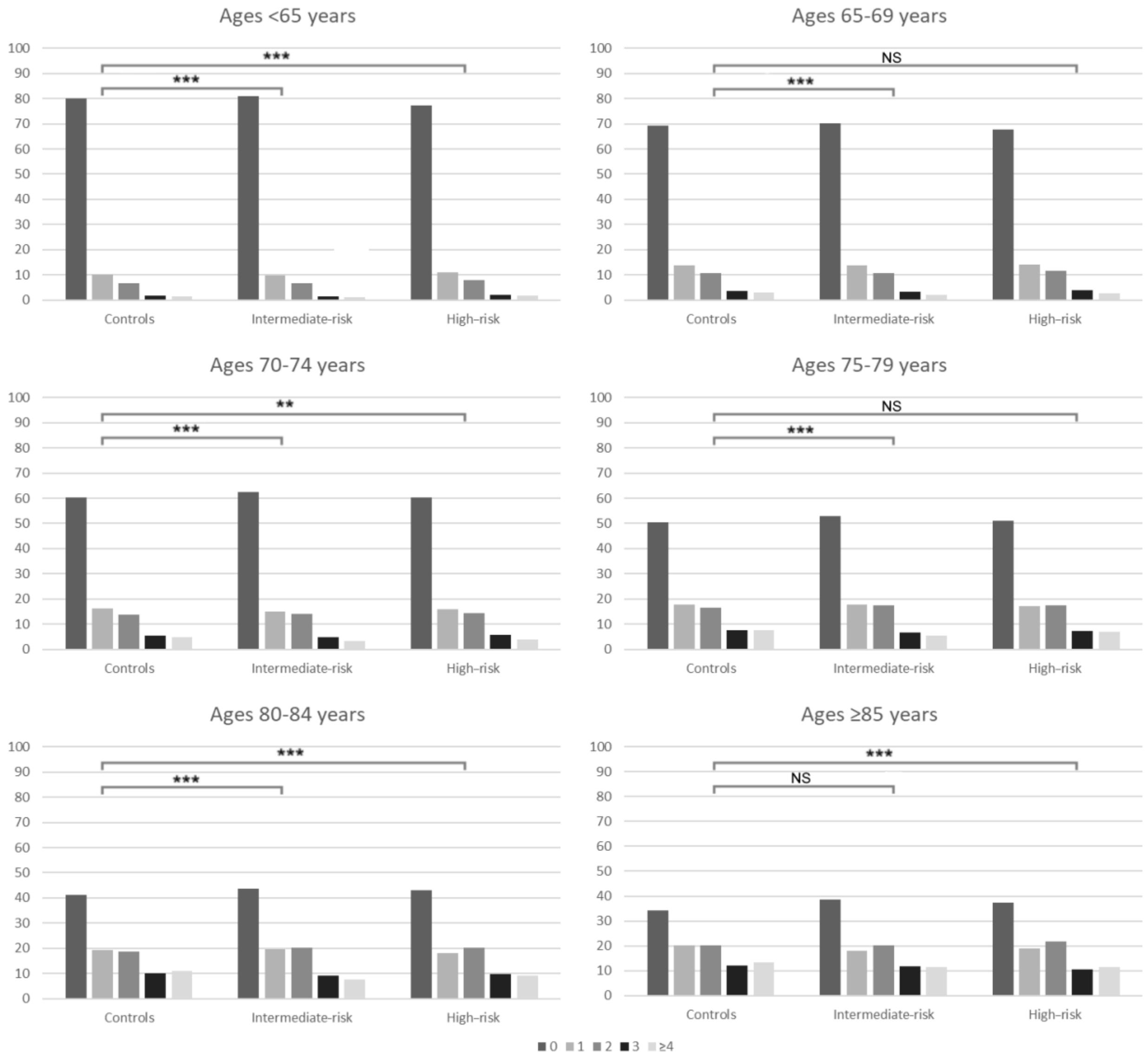

Within each age group, the Charlson Comorbidity Index distribution in men with intermediate- and high-risk localized prostate cancer was compared to the control population available in PcBaSe using χ^2-tests.

Supplementary Figure 2. Drug Comorbidity Index in intermediate- and high-risk prostate cancer cases compared to controls

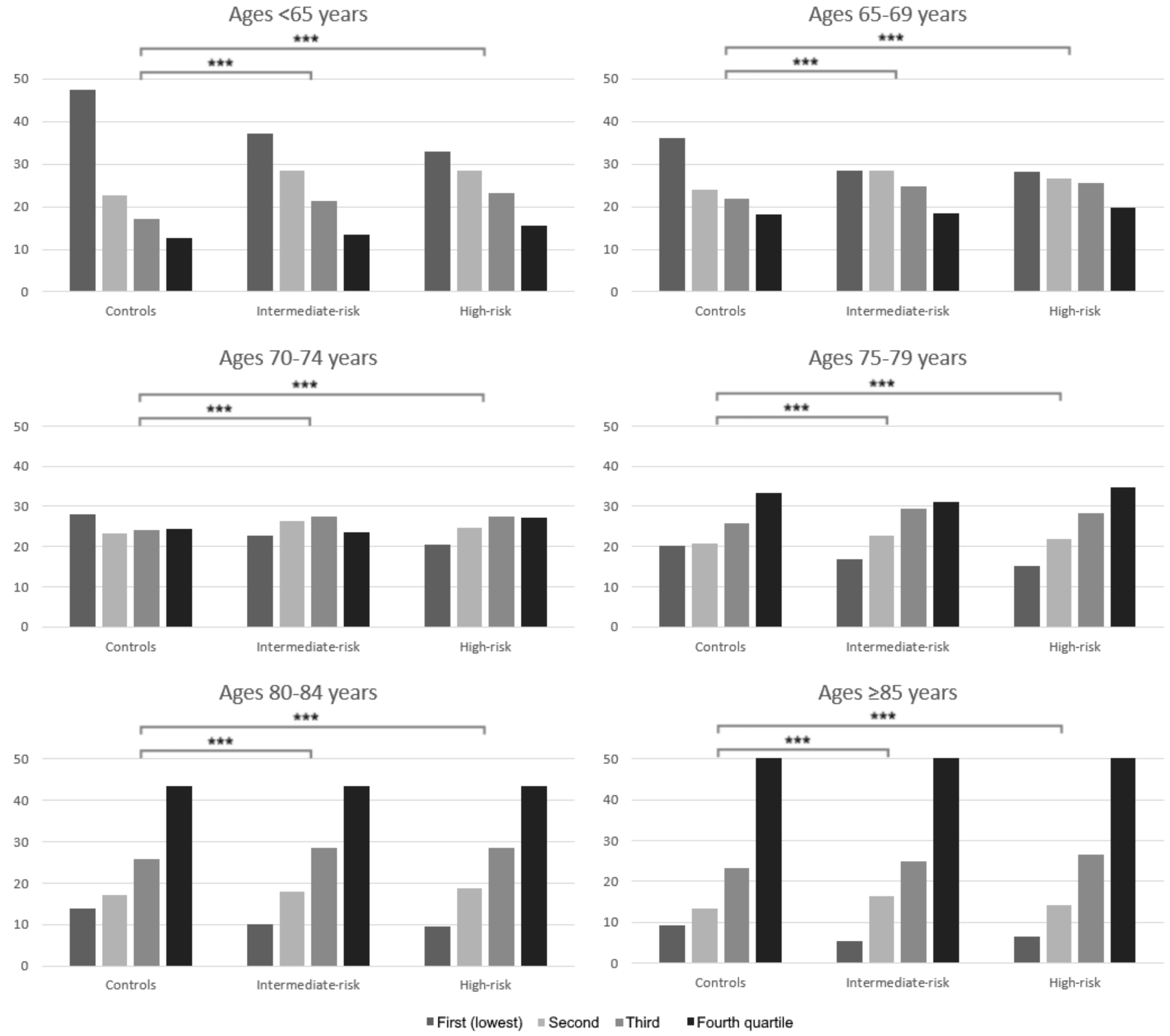

Within each age group, the Drug Comorbidity Index distribution in men with intermediate- and high-risk localized prostate cancer was compared to the control population available in PcBaSe using χ^2-tests.
