# Supplementary material for: Time trends in the use of curative treatment in men 70 years and older with nonmetastatic prostate cancer
Source: Acta Oncol. 2024 Mar 20;63:26189. doi: 10.2340/1651-226X.2024.26189 (PMC11332516; doi:10.2340/1651-226X.2024.26189)
Supplement: Time trends in the use of curative treatment in men 70 years and older with nonmetastatic prostate cancer [file AO-63-26189-s2.pdf]

Supplementary material has been published as submitted. It has not been copyedited or typeset by Acta Oncologica.

Supplementary Table 1. Characteristics of intermediate-risk prostate cancer patients

**Age at diagnosis**	**<65y**		**65-69y**		**70-74y**		**75-79y**		**80-84y**		**≥85y**		**Total**	
	**No.**	**%**	**No.**	**%**	**No.**	**%**	**No.**	**%**	**No.**	**%**	**No.**	**%**	**No.**	**%**
**Total (row percentage)**	13,289	(30.7)	11,159	(25.8)	9,743	(22.5)	5,932	(13.7)	2,365	(5.5)	741	(1.7)	43,229	(100.0)
**Country of birth**														
Sweden	11,965	90.0	10,209	91.5	8,822	90.5	5,341	90.0	2,151	91.0	677	91.4	39,165	90.6
other Nordic country	436	3.3	390	3.5	411	4.2	279	4.7	96	4.1	21	2.8	1,633	3.8
Non-Nordic country	887	6.7	559	5.0	510	5.2	312	5.3	118	5.0	43	5.8	2,429	5.6
Missing	1	0.0	1	0.0	0	0.0	0	0.0	0	0.0	0	0.0	2	0.0
**Education level**														
Elementary	2,607	19.6	2,935	26.3	3,119	32.0	2,177	36.7	1,056	44.7	352	47.5	12,246	28.3
Gymnasium	6,142	46.2	4,728	42.4	3,904	40.1	2,315	39.0	803	34.0	232	31.3	18,124	41.9
University	4,540	34.2	3,496	31.3	2,720	27.9	1,440	24.3	506	21.4	157	21.2	12,859	29.7
**Marital status at diagnosis**														
Unmarried	2,579	19.4	1,386	12.4	828	8.5	396	6.7	155	6.6	38	5.1	5,382	12.4
Married	8,199	61.7	7,532	67.5	6,746	69.2	4,118	69.4	1,558	65.9	405	54.7	28,558	66.1
Divorced	2,282	17.2	1,824	16.3	1,550	15.9	824	13.9	255	10.8	67	9.0	6,802	15.7
Widower	219	1.6	409	3.7	611	6.3	594	10.0	397	16.8	231	31.2	2,461	5.7
Missing	10	0.1	8	0.1	8	0.1	0	0.0	0	0.0	0	0.0	26	0.1
**Year of diagnosis**														
2008-2011	3,855	29.0	2,993	26.8	2,364	24.3	1,428	24.1	696	29.4	244	32.9	11,580	26.8
2012-2014	3,044	22.9	2,832	25.4	1,932	19.8	1,064	17.9	423	17.9	149	20.1	9,444	21.8
2015-2017	3,057	23.0	2,713	24.3	2,684	27.5	1,545	26.0	564	23.8	181	24.4	10,744	24.9
2018-2020	3,333	25.1	2,621	23.5	2,763	28.4	1,895	31.9	682	28.8	167	22.5	11,461	26.5
**Mode of detection**														
Check-up	8,927	67.2	6,954	62.3	5,564	57.1	3,058	51.6	974	41.2	220	29.7	25,697	59.4
LUTS	2,544	19.1	2,620	23.5	2,691	27.6	1,947	32.8	979	41.4	348	47.0	11,129	25.7
Other symptoms	1,482	11.2	1,224	11.0	1,205	12.4	778	13.1	347	14.7	157	21.2	5,193	12.0
Missing	336	2.5	361	3.2	283	2.9	149	2.5	65	2.7	16	2.2	1,210	2.8

**Charlson Comorbidity Index**														
0	10,774	81.1	7,835	70.2	6,076	62.4	3,139	52.9	1,030	43.6	287	38.7	29,141	67.4
1	1,278	9.6	1,525	13.7	1,476	15.1	1,053	17.8	467	19.7	133	17.9	5,932	13.7
2	892	6.7	1,206	10.8	1,383	14.2	1,031	17.4	477	20.2	149	20.1	5,138	11.9
3	197	1.5	363	3.3	478	4.9	395	6.7	212	9.0	88	11.9	1,733	4.0
4 or more	148	1.1	230	2.1	330	3.4	314	5.3	179	7.6	84	11.3	1,285	3.0
**Drug Comorbidity Index**														
1st quartile (least)	4,919	37.0	3,170	28.4	2,203	22.6	1,003	16.9	238	10.1	39	5.3	11,572	26.8
2nd quartile	3,770	28.4	3,177	28.5	2,563	26.3	1,346	22.7	422	17.8	121	16.3	11,399	26.4
3rd quartile	2,839	21.4	2,772	24.8	2,677	27.5	1,737	29.3	676	28.6	185	25.0	10,886	25.2
4th quartile	1,761	13.3	2,040	18.3	2,300	23.6	1,846	31.1	1,029	43.5	396	53.4	9,372	21.7
**Tumor size at diagnosis**														
T0	9,005	67.8	7,350	65.9	5,861	60.2	3,222	54.3	1,103	46.6	317	42.8	26,858	62.1
T1	4,256	32.0	3,782	33.9	3,862	39.6	2,695	45.4	1,252	52.9	419	56.5	16,266	37.6
T2	28	0.2	27	0.2	20	0.2	15	0.3	10	0.4	5	0.7	105	0.2
**PSA at diagnosis**														
<10	9,335	70.2	7,447	66.7	5,705	58.6	3,022	50.9	859	36.3	192	25.9	26,560	61.4
10-19.9	3,886	29.2	3,645	32.7	3,934	40.4	2,807	47.3	1,437	60.8	502	67.7	16,211	37.5
Missing	68	0.5	67	0.6	104	1.1	103	1.7	69	2.9	47	6.3	458	1.1
**Gleason score at diagnosis**														
1-6	1,850	13.9	1,714	15.4	1,620	16.6	1,048	17.7	452	19.1	132	17.8	6,816	15.8
7	11,409	85.9	9,421	84.4	8,080	82.9	4,836	81.5	1,853	78.4	553	74.6	36,152	83.6
Missing	30	0.2	24	0.2	43	0.4	48	0.8	60	2.5	56	7.6	260	0.6
**Primary treatment planned**														
Curative	11,099	83.5	8,545	76.6	6,274	64.4	2,088	35.2	161	6.8	4	0.5	28,171	65.2
Conservative	1,946	14.6	2,258	20.2	2,817	28.9	2,649	44.7	1,340	56.7	398	53.7	11,408	26.4
ADT	67	0.5	175	1.6	500	5.1	1,083	18.3	805	34.0	312	42.1	2,942	6.8
Not registered	177	1.3	181	1.6	152	1.6	112	1.9	59	2.5	27	3.6	708	1.6

LUTS: lower urinary tract symptoms, PSA: prostate-specific antigen, ADT: androgen deprivation therapy.

Supplementary Table 2. Characteristics of high-risk prostate cancer patients

Age at diagnosis	<65y		65-69y		70-74y		75-79y		80-84y		≥85y		Total	
	No.	%	No.	%	No.	%	No.	%	No.	%	No.	%	No.	%
**Total (row percentage)**	4,316	(16.1)	4,873	(18.2)	5,666	(21.1)	5,436	(20.2)	4,044	(15.1)	2,510	(9.3)	26,845	(100.0)
**Country of birth**														
Sweden	3,868	89.6	4,414	90.6	5,154	91.0	4,895	90.0	3,701	91.5	2,365	94.2	24,397	90.9
other Nordic country	140	3.2	200	4.1	234	4.1	248	4.6	151	3.7	51	2.0	1,024	3.8
Non-Nordic country	308	7.1	259	5.3	278	4.9	293	5.4	192	4.7	94	3.7	1,424	5.3
**Education level**														
Elementary	932	21.6	1,431	29.4	2,023	35.7	2,264	41.6	1,891	46.8	1,183	47.1	9,724	36.2
Gymnasium	2,027	47.0	2,044	41.9	2,214	39.1	1,963	36.1	1,328	32.8	837	33.3	10,413	38.8
University	1,357	31.4	1,398	28.7	1,429	25.2	1,209	22.2	825	20.4	490	19.5	6,708	25.0
**Marital status at diagnosis**														
Unmarried	952	22.1	684	14.0	622	11.0	428	7.9	254	6.3	99	3.9	3,039	11.3
Married	2,537	58.8	3,143	64.5	3,744	66.1	3,557	65.4	2,559	63.3	1,385	55.2	16,925	63.0
Divorced	750	17.4	849	17.4	943	16.6	810	14.9	461	11.4	195	7.8	4,008	14.9
Widower	76	1.8	192	3.9	355	6.3	639	11.8	770	19.0	831	33.1	2,863	10.7
Missing	1	0.0	5	0.1	2	0.0	2	0.0	0	0.0	0	0.0	10	0.0
**Year of diagnosis**														
2008-2011	1,526	35.4	1,578	32.4	1,651	29.1	1,745	32.1	1,369	33.9	840	33.5	8,709	32.4
2012-2014	980	22.7	1,226	25.2	1,210	21.4	1,051	19.3	878	21.7	536	21.4	5,881	21.9
2015-2017	943	21.8	1,085	22.3	1,397	24.7	1,284	23.6	918	22.7	609	24.3	6,236	23.2
2018-2020	867	20.1	984	20.2	1,408	24.8	1,356	24.9	879	21.7	525	20.9	6,019	22.4
**Mode of detection**														
Check-up	2,445	56.6	2,616	53.7	2,798	49.4	2,287	42.1	1,366	33.8	662	26.4	12,174	45.3
LUTS	1,143	26.5	1,471	30.2	1,838	32.4	2,109	38.8	1,778	44.0	1,225	48.8	9,564	35.6
Other symptoms	617	14.3	660	13.5	851	15.0	897	16.5	786	19.4	559	22.3	4,370	16.3
Missing	111	2.6	126	2.6	179	3.2	143	2.6	114	2.8	64	2.5	737	2.7
**Charlson Comorbidity Index**														
0	3,338	77.3	3,296	67.6	3,410	60.2	2,776	51.1	1,740	43.0	935	37.3	15,495	57.7
1	474	11.0	688	14.1	903	15.9	930	17.1	735	18.2	478	19.0	4,208	15.7
2	347	8.0	569	11.7	814	14.4	952	17.5	813	20.1	545	21.7	4,040	15.0
3	82	1.9	189	3.9	315	5.6	393	7.2	392	9.7	266	10.6	1,637	6.1
4 or more	75	1.7	131	2.7	224	4.0	385	7.1	364	9.0	286	11.4	1,465	5.5
**Drug Comorbidity Index**														
1st quartile (least)	1,423	33.0	1,368	28.1	1,167	20.6	826	15.2	384	9.5	164	6.5	5,332	19.9

2nd quartile	1,231	28.5	1,293	26.5	1,405	24.8	1,186	21.8	756	18.7	357	14.2	6,228	23.2
3rd quartile	997	23.1	1,246	25.6	1,554	27.4	1,534	28.2	1,151	28.5	668	26.6	7,150	26.6
4th quartile	665	15.4	966	19.8	1,540	27.2	1,890	34.8	1,753	43.3	1,321	52.6	8,135	30.3
**Tumor size at diagnosis**														
T1	1,551	35.9	1,640	33.7	1,839	32.5	1,496	27.5	850	21.0	446	17.8	7,822	29.1
T2	1,506	34.9	1,722	35.3	2,082	36.7	2,002	36.8	1,517	37.5	909	36.2	9,738	36.3
T3	1,176	27.2	1,452	29.8	1,661	29.3	1,851	34.1	1,604	39.7	1,094	43.6	8,838	32.9
Missing	83	1.9	59	1.2	84	1.5	87	1.6	73	1.8	61	2.4	447	1.7
**PSA at diagnosis**														
<10	1,706	39.5	1,907	39.1	2,016	35.6	1,530	28.1	711	17.6	301	12.0	8,171	30.4
10-19.9	784	18.2	978	20.1	1,242	21.9	1,319	24.3	1,027	25.4	549	21.9	5,899	22.0
20-49.9	1,802	41.8	1,966	40.3	2,356	41.6	2,523	46.4	2,217	54.8	1,556	62.0	12,420	46.3
Missing	24	0.6	22	0.5	52	0.9	64	1.2	89	2.2	104	4.1	355	1.3
**Gleason score at diagnosis**														
1-6	561	13.0	554	11.4	560	9.9	499	9.2	352	8.7	166	6.6	2,692	10.0
7	1,312	30.4	1,486	30.5	1,714	30.3	1,707	31.4	1,278	31.6	750	29.9	8,247	30.7
8-10	2,417	56.0	2,801	57.5	3,332	58.8	3,121	57.4	2,211	54.7	1,323	52.7	15,205	56.6
Missing	26	0.6	32	0.7	60	1.1	109	2.0	203	5.0	271	10.8	701	2.6
**Primary treatment planned**														
Curative	3,837	88.9	4,060	83.3	4,139	73.0	2,301	42.3	412	10.2	28	1.1	14,777	55.0
Conservative	235	5.4	323	6.6	508	9.0	775	14.3	713	17.6	497	19.8	3,051	11.4
ADT	160	3.7	413	8.5	920	16.2	2,230	41.0	2,816	69.6	1,906	75.9	8,445	31.5
Not registered	84	1.9	77	1.6	99	1.7	130	2.4	103	2.5	79	3.1	572	2.1

LUTS: lower urinary tract symptoms, PSA: prostate-specific antigen, ADT: androgen deprivation therapy

Supplementary Table 3. Staging of high-risk prostate cancer 2012-2020

**High-risk prostate cancer**			
**Abdominal imaging, yes vs no**	**yes**	**no**	**OR (95% CI)**
<65 y	1,485 (53%)	1,305 (47%)	0.95 (0.85-1.06)
65-69 y	1,779 (54%)	1,516 (46%)	1.00 (reference)
70-74 y	2,161 (54%)	1,854 (46%)	0.93 (0.84-1.02)
75-79 y	1,623 (44%)	2,068 (56%)	0.60 (0.54-0.67)
80-84 y	539 (20%)	2,136 (80%)	0.20 (0.17-0.22)
≥85 y	140 (8%)	1,530 (92%)	0.07 (0.06-0.09)
**Bone imaging, yes vs no**	**yes**	**no**	**OR (95% CI)**
<65 y	1,980 (71%)	810 (29%)	0.92 (0.82-1.04)
65-69 y	2,385 (72%)	910 (28%)	1.00 (reference)
70-74 y	2,995 (75%)	1,020 (25%)	1.09 (0.98-1.21)
75-79 y	2,552 (69%)	1,139 (31%)	0.84 (0.75-0.93)
80-84 y	1,390 (52%)	1,285 (48%)	0.42 (0.38-0.47)
≥85 y	631 (38%)	1,039 (62%)	0.24 (0.21-0.27)

Odds ratios adjusted for country of birth, education level, marital status, year of diagnosis, Charlson Comorbidity Index and Drug Comorbidity Index. CI: confidence interval, OR: odds ratio.

Supplementary Table 4. Primary treatment, intermediate- and high-risk prostate cancer patients with Charlson Comorbidity Index 0

			RRR comparing curative to conservative and ADT		
**Intermediate-risk prostate cancer**			**Model 1**	**Model 2**	**Model 3**
**Curative vs conservative**	**Curative**	**Cons.**	**RRR (95% CI)**	**RRR (95% CI)**	**RRR (95% CI)**
<65 y	9,060 (85%)	1,549 (15%)	1.43 (1.32-1.54)	1.48 (1.36-1.61)	1.45 (1.33-1.58)
65-69 y	6,168 (80%)	1,488 (19%)	1.00 (reference)	1.00 (reference)	1.00 (reference)
70-74 y	4,088 (68%)	1,677 (28%)	0.58 (0.54-0.63)	0.54 (0.49-0.59)	0.54 (0.50-0.59)
75-79 y	1,210 (39%)	1,364 (44%)	0.21 (0.19-0.23)	0.16 (0.14-0.18)	0.17 (0.15-0.18)
80-84 y	86 (9%)	589 (59%)	0.03 (0.03-0.04)	0.02 (0.02-0.03)	0.02 (0.02-0.03)
≥85 y	0 (0%)	155 (56%)	N/A	N/A	N/A
**Curative vs ADT**	**Curative**	**ADT**			
<65 y	9,060 (85%)	37 (0%)	2.77 (1.85-4.14)	2.83 (1.87-4.28)	2.71 (1.79-4.09)
65-69 y	6,168 (80%)	67 (1%)	1.00 (reference)	1.00 (reference)	1.00 (reference)
70-74 y	4,088 (68%)	232 (4%)	0.18 (0.14-0.24)	0.19 (0.14-0.25)	0.19 (0.15-0.26)
75-79 y	1,210 (39%)	524 (17%)	0.02 (0.02-0.03)	0.02 (0.02-0.03)	0.03 (0.02-0.03)
80-84 y	86 (9%)	328 (33%)	0.00 (0.00-0.00)	0.00 (0.00-0.00)	0.00 (0.00-0.00)
≥85 y	0 (0%)	122 (44%)	N/A	N/A	N/A
**High-risk prostate cancer**			**Model 1**	**Model 2**	**Model 3**
**Curative vs conservative**	**Curative**	**Cons.**	**RRR (95% CI)**	**RRR (95% CI)**	**RRR (95% CI)**
<65 y	3,011 (92%)	162 (5%)	1.44 (1.16-1.79)	1.77 (1.39-2.26)	1.74 (1.36-2.23)
65-69 y	2,827 (87%)	204 (6%)	1.00 (reference)	1.00 (reference)	1.00 (reference)
70-74 y	2,662 (79%)	263 (8%)	0.66 (0.54-0.80)	0.58 (0.47-0.72)	0.61 (0.49-0.75)
75-79 y	1,330 (49%)	389 (14%)	0.20 (0.16-0.24)	0.14 (0.11-0.17)	0.14 (0.12-0.18)
80-84 y	207 (12%)	303 (18%)	0.04 (0.03-0.05)	0.02 (0.02-0.03)	0.02 (0.02-0.03)
≥85 y	13 (1%)	170 (19%)	0.00 (0.00-0.01)	0.00 (0.00-0.00)	0.00 (0.00-0.00)
**Curative vs ADT**	**Curative**	**ADT**			
<65 y	3,011 (92%)	101 (3%)	2.51 (1.96-3.21)	2.50 (1.94-3.22)	2.47 (1.91-3.18)
65-69 y	2,827 (87%)	216 (7%)	1.00 (reference)	1.00 (reference)	1.00 (reference)
70-74 y	2,662 (79%)	443 (13%)	0.40 (0.33-0.47)	0.39 (0.33-0.47)	0.41 (0.34-0.49)
75-79 y	1,330 (49%)	1,009 (37%)	0.07 (0.06-0.09)	0.07 (0.06-0.08)	0.07 (0.06-0.09)
80-84 y	207 (12%)	1,190 (70%)	0.01 (0.01-0.01)	0.01 (0.01-0.01)	0.01 (0.01-0.01)
≥85 y	13 (1%)	723 (80%)	0.00 (0.00-0.00)	0.00 (0.00-0.00)	0.00 (0.00-0.00)

Model 1: Adjusted for marital status, education, country of birth, year of diagnosis
Model 2: Also adjusted for tumor size, Gleason sum, PSA-level
Model 3: Also adjusted for Drug Comorbidity Index
ADT: androgen deprivation therapy, CI: confidence interval, Cons: conservative, RRR: relative risk ratio.

Supplementary Table 5. Primary treatment, intermediate- and high-risk prostate cancer patients with Charlson Comorbidity Index 0 and Drug Comorbidity Index 1st quartile

			RRR comparing curative to conservative and ADT	
**Intermediate-risk prostate cancer**			**Model 1**	**Model 2**
**Curative vs conservative**	**Curative**	**Cons.**	**RRR (95% CI)**	**RRR (95% CI)**
<65 y	3,857 (86%)	627 (14%)	1.39 (1.22-1.59)	1.41 (1.22-1.62)
65-69 y	2,197 (81%)	491 (18%)	1.00 (reference)	1.00 (reference)
70-74 y	1,342 (74%)	430 (24%)	0.70 (0.60-0.81)	0.63 (0.54-0.74)
75-79 y	331 (44%)	325 (43%)	0.23 (0.19-0.27)	0.17 (0.14-0.21)
80-84 y	28 (15%)	104 (57%)	0.06 (0.04-0.09)	0.03 (0.02-0.05)
≥85 y	0 (0%)	15 (48%)	N/A	N/A
**Curative vs ADT**	**Curative**	**ADT**		
<65 y	3,857 (86%)	8 (0%)	3.63 (1.55-8.52)	3.57 (1.52-8.38)
65-69 y	2,197 (81%)	16 (1%)	1.00 (reference)	1.00 (reference)
70-74 y	1,342 (74%)	40 (2%)	0.22 (0.12-0.40)	0.24 (0.13-0.43)
75-79 y	331 (44%)	103 (14%)	0.02 (0.01-0.04)	0.02 (0.01-0.04)
80-84 y	28 (15%)	49 (27%)	0.00 (0.00-0.01)	0.00 (0.00-0.01)
≥85 y	0 (0%)	16 (52%)	N/A	N/A
**High-risk prostate cancer**			**Model 1**	**Model 2**
**Curative vs conservative**	**Curative**	**Cons.**	**RRR (95% CI)**	**RRR (95% CI)**
<65 y	1,196 (93%)	60 (5%)	1.37 (0.95-1.98)	1.57 (1.04-2.39)
65-69 y	1,048 (88%)	66 (6%)	1.00 (reference)	1.00 (reference)
70-74 y	814 (83%)	59 (6%)	0.78 (0.54-1.13)	0.70 (0.46-1.07)
75-79 y	355 (56%)	75 (12%)	0.23 (0.16-0.34)	0.14 (0.09-0.22)
80-84 y	41 (16%)	46 (17%)	0.04 (0.02-0.06)	0.02 (0.01-0.04)
≥85 y	3 (3%)	14 (12%)	0.01 (0.00-0.03)	0.01 (0.00-0.03)
**Curative vs ADT**	**Curative**	**ADT**		
<65 y	1,196 (93%)	34 (3%)	2.55 (1.67-3.91)	2.33 (1.51-3.61)
65-69 y	1,048 (88%)	72 (6%)	1.00 (reference)	1.00 (reference)
70-74 y	814 (83%)	108 (11%)	0.45 (0.32-0.62)	0.42 (0.30-0.59)
75-79 y	355 (56%)	209 (33%)	0.08 (0.06-0.11)	0.08 (0.05-0.11)
80-84 y	41 (16%)	176 (67%)	0.01 (0.00-0.01)	0.01 (0.00-0.01)
≥85 y	3 (3%)	96 (85%)	0.00 (0.00-0.00)	0.00 (0.00-0.00)

Model 1: Adjusted for marital status, education, country of birth, year of diagnosis
Model 2: Also adjusted for tumor size, Gleason sum, PSA-level
ADT: androgen deprivation therapy, CI: confidence interval, Cons: conservative, RRR: relative risk ratio.

Supplementary table 6. Primary treatment planned for intermediate- and high-risk prostate cancer patients, by calendar period of diagnosis

**Intermediate-risk prostate cancer**	**2008-2016**			**2017-2020**		
**Curative vs conservative**	**Curative**	**Cons.**	**RRR (95% CI)**	**Curative**	**Cons.**	**RRR (95% CI)**
<65 y	7,501 (86%)	1,208 (14%)	1.68 (1.53-1.84)	3,598 (83%)	738 (17%)	1.20 (1.06-1.37)
65-69 y	5,855 (78%)	1,552 (21%)	1.00 (reference)	2,690 (79%)	706 (21%)	1.00 (reference)
70-74 y	3,718 (62%)	1,870 (31%)	0.49 (0.45-0.53)	2,556 (71%)	947 (26%)	0.69 (0.61-0.79)
75-79 y	904 (26%)	1,700 (49%)	0.11 (0.10-0.12)	1,184 (50%)	949 (40%)	0.29 (0.26-0.34)
80-84 y	59 (4%)	852 (59%)	0.01 (0.01-0.02)	102 (12%)	488 (58%)	0.04 (0.03-0.06)
≥85 y	1 (0%)	272 (56%)	0.00 (0.00-0.01)	3 (1%)	126 (56%)	0.00 (0.00-0.01)
**Curative vs ADT**	**Curative**	**ADT**	**RRR (95% CI)**	**Curative**	**ADT**	**RRR (95% CI)**
<65 y	7,501 (86%)	50 (1%)	3.46 (2.48-4.82)	3,598 (83%)	17 (0%)	1.98 (1.07-3.65)
65-69 y	5,855 (78%)	148 (2%)	1.00 (reference)	2,690 (79%)	27 (1%)	1.00 (reference)
70-74 y	3,718 (62%)	413 (7%)	0.25 (0.20-0.30)	2,556 (71%)	87 (2%)	0.34 (0.22-0.52)
75-79 y	904 (26%)	852 (25%)	0.03 (0.02-0.04)	1,184 (50%)	231 (10%)	0.06 (0.04-0.09)
80-84 y	59 (4%)	546 (38%)	0.00 (0.00-0.01)	102 (12%)	259 (31%)	0.01 (0.00-0.01)
≥85 y	1 (0%)	215 (44%)	0.00 (0.00-0.00)	3 (1%)	97 (43%)	0.00 (0.00-0.00)
**High-risk prostate cancer**	**2008-2016**			**2017-2020**		
**Curative vs conservative**	**Curative**	**Cons.**	**RRR (95% CI)**	**Curative**	**Cons.**	**RRR (95% CI)**
<65 y	2,768 (90%)	160 (5%)	1.69 (1.34-2.15)	1,069 (92%)	75 (6%)	1.25 (0.84-1.87)
65-69 y	2,897 (83%)	231 (7%)	1.00 (reference)	1,163 (89%)	92 (7%)	1.00 (reference)
70-74 y	2,586 (70%)	360 (10%)	0.49 (0.40-0.60)	1,553 (84%)	148 (8%)	0.79 (0.56-1.10)
75-79 y	1,108 (31%)	610 (17%)	0.10 (0.08-0.12)	1,193 (67%)	165 (9%)	0.43 (0.31-0.61)
80-84 y	137 (5%)	528 (19%)	0.01 (0.01-0.02)	275 (24%)	185 (16%)	0.07 (0.05-0.10)
≥85 y	10 (1%)	349 (21%)	0.00 (0.00-0.00)	18 (2%)	148 (20%)	0.01 (0.00-0.01)
**Curative vs ADT**	**Curative**	**ADT**	**RRR (95% CI)**	**Curative**	**ADT**	**RRR (95% CI)**
<65 y	2,768 (90%)	140 (5%)	2.53 (2.04-3.13)	1,069 (92%)	20 (2%)	2.12 (1.24-3.62)
65-69 y	2,897 (83%)	360 (10%)	1.00 (reference)	1,163 (89%)	53 (4%)	1.00 (reference)
70-74 y	2,586 (70%)	765 (21%)	0.41 (0.35-0.47)	1,553 (84%)	155 (8%)	0.55 (0.39-0.78)
75-79 y	1,108 (31%)	1810 (51%)	0.07 (0.06-0.08)	1,193 (67%)	420 (24%)	0.16 (0.11-0.21)
80-84 y	137 (5%)	2108 (76%)	0.01 (0.01-0.01)	275 (24%)	708 (61%)	0.02 (0.02-0.03)
≥85 y	10 (1%)	1335 (79%)	0.00 (0.00-0.00)	18 (2%)	571 (78%)	0.00 (0.00-0.00)

Adjusted for marital status, education, country of birth, year of diagnosis, tumor size, Gleason sum, PSA-level, Charlson Comorbidity Index and Drug Comorbidity Index. ADT: androgen deprivation therapy, CI: confidence interval, Cons: conservative, PSA: Prostate-specific antigen; RRR: relative risk ratio.
